# Evaluating the accuracy of cerebrovascular computational fluid dynamics modeling through time-resolved experimental validation

**DOI:** 10.1038/s41598-024-58925-8

**Published:** 2024-04-08

**Authors:** Claudio A. Luisi, Tom L. Witter, Omid Nikoubashman, Martin Wiesmann, Ulrich Steinseifer, Michael Neidlin

**Affiliations:** 1 Department of Cardiovascular Engineering, Institute of Applied Medical Engineering, Medical Faculty, RWTH Aachen University, Pauwelsstr. 20, 52074 Aachen, Germany; 2 Clinic for Diagnostic and Interventional Neuroradiology, Medical Faculty, RWTH Aachen University, Pauwelstr. 30, 52074 Aachen, Germany

**Keywords:** Biomedical engineering, Stroke, Computational science, Fluid dynamics

## Abstract

Accurate modeling of cerebral hemodynamics is crucial for better understanding the hemodynamics of stroke, for which computational fluid dynamics (CFD) modeling is a viable tool to obtain information. However, a comprehensive study on the accuracy of cerebrovascular CFD models including both transient arterial pressures and flows does not exist. This study systematically assessed the accuracy of different outlet boundary conditions (BCs) comparing CFD modeling and an in-vitro experiment. The experimental setup consisted of an anatomical cerebrovascular phantom and high-resolution flow and pressure data acquisition. The CFD model of the same cerebrovascular geometry comprised five sets of stationary and transient BCs including established techniques and a novel BC, the phase modulation approach. The experiment produced physiological hemodynamics consistent with reported clinical results for total cerebral blood flow, inlet pressure, flow distribution, and flow pulsatility indices (PI). The in-silico model instead yielded time-dependent deviations between 19–66% for flows and 6–26% for pressures. For cerebrovascular CFD modeling, it is recommended to avoid stationary outlet pressure BCs, which caused the highest deviations. The Windkessel and the phase modulation BCs provided realistic flow PI values and cerebrovascular pressures, respectively. However, this study shows that the accuracy of current cerebrovascular CFD models is limited.

## Introduction

Stroke is the second major contributor to global burden after the age of 50^1^ with the majority of stroke cases being ischemic^2^, while the remaining proportion are hemorrhagic strokes. For better understanding the pathophysiology of acute ischemic stroke and cerebral aneurysm formation, which potentially leads to hemorrhagic stroke, as well as for improving endovascular treatments (EVT) thereof, knowledge on cerebral hemodynamics is crucial. In order to enhance this knowledge, accurate information on both flows as well as pressures within the intracranial arteries is mandatory. However, attaining time-resolved information on both cerebrovascular flows and pressures is challenging, as evidenced by several in-vivo, in-vitro and in-silico studies that focused on analyzing cerebral hemodynamics.

In-vivo measurements of intracranial blood flow velocities using phase-contrast magnetic resonance imaging (PC-MRI) have been performed in several investigations since the early studies of this technique^3^. For instance, Correia de Verdier and Wikström investigated cerebral blood flow and flow pulsatility index (PI) for 30 subjects via 2D PC-MRI^4^. Zarrinkoob et al. determined age-dependent blood flow distribution and PI of the cerebral arteries via 2D PC-MRI of 94 subjects^5,6^. In some 4D Flow MRI studies, intracranial velocities were determined via 3T for 25 subjects^7^ and both 3T and 7T for 5 subjects^8^. However, PC-MRI has limited temporal and spatial resolution^7,8^, making transient cerebral hemodynamics challenging to investigate in vivo. Moreover, in order to reconstruct one mean cardiac cycle from all the cardiac cycles of the acquisition duration, cardiac synchronization is needed. Additionally, despite recent advances in 4D Flow MRI relative pressure estimation^9^, it is currently not feasible to estimate absolute pressure non-invasively^10^.

In-vitro studies were conducted in order to overcome the limitations of the in-vivo measurements and allowing for better understanding of flows and pressures within the cerebral arteries in physiological and pathological cases, as well as in case of EVT. An established EVT technique for the treatment of ischemic stroke is thrombectomy, the mechanical removal of blood clots from the occluded vessel. It was indicated that the force required during thrombectomy in cerebral vessels is substantially influenced by the arterial pressure gradient across the clot^11^; hence, physiologically relevant pressures have to be accurately modeled. Few studies investigated transient flows and pressures in patient-specific compliant phantoms of the cerebral arteries with complete Circle of Willis (CoW) anatomies^12,13^. Some limitations of the in-vitro studies were the difficulty in creating compliant phantom models for small arteries and the costs of such models^14^; thus, in-vitro investigations are not yet a viable option for large-scale studies. However, in order to better understand the physiology, pathophysiology, and finally improve the therapy, insight into local cerebral hemodynamics is needed.

Computational fluid dynamics (CFD) of 3D models allow insight into local hemodynamics and account for the complex anatomy of the cerebral arteries, but the reliability of such models depends on the scale of validation. Since the acquisition of detailed in-vivo data is challenging, it is crucial to validate in-silico models with results from in-vitro models^11^. It is known that boundary conditions (BCs) of the in-silico models strongly influence the hemodynamic results. Some studies discussed the impact of physiological inlet BCs of cerebral arteries and highlighted the importance of outlet BCs^15^, as they are substantial contributors to uncertainty of solutions with multiple outlets^15,16^. For the transient investigation of physiological cerebral hemodynamics in complete CoW anatomies, researchers used different pressure-based outlet BCs: stationary zero pressure^15^, generic transient pressure waveforms^17^, and multiscale models^18,19^. In another approach, outlet flows were directly imposed^15,20^. However, a comprehensive study on the accuracy of cerebrovascular CFD models assessing both transient arterial pressures as well as flows has not yet been performed. Moreover, such studies require well-calibrated in-vitro models, as in-vivo acquisitions of simultaneous pressure and flow measurements within cerebral arteries are hardly possible^19^.

To close this gap, we performed a combined in-vitro and in-silico study acquiring and analyzing transient cerebral pressures and flows. Our objective was to evaluate cerebrovascular CFD modeling accuracy and identify the needed modeling detail for the study of cerebral hemodynamics. To achieve this objective, we performed an in-silico investigation of established as well as new outlet BCs and determined the time-dependent deviation of flows and pressures through validation with a time-resolved experiment on a phantom model of the same cerebrovascular anatomy. The experiment was calibrated to in-vivo data including flow PI values for generating a physiological and clinically plausible case.

## Materials and methods

### In-vitro setup

**Figure 1:**
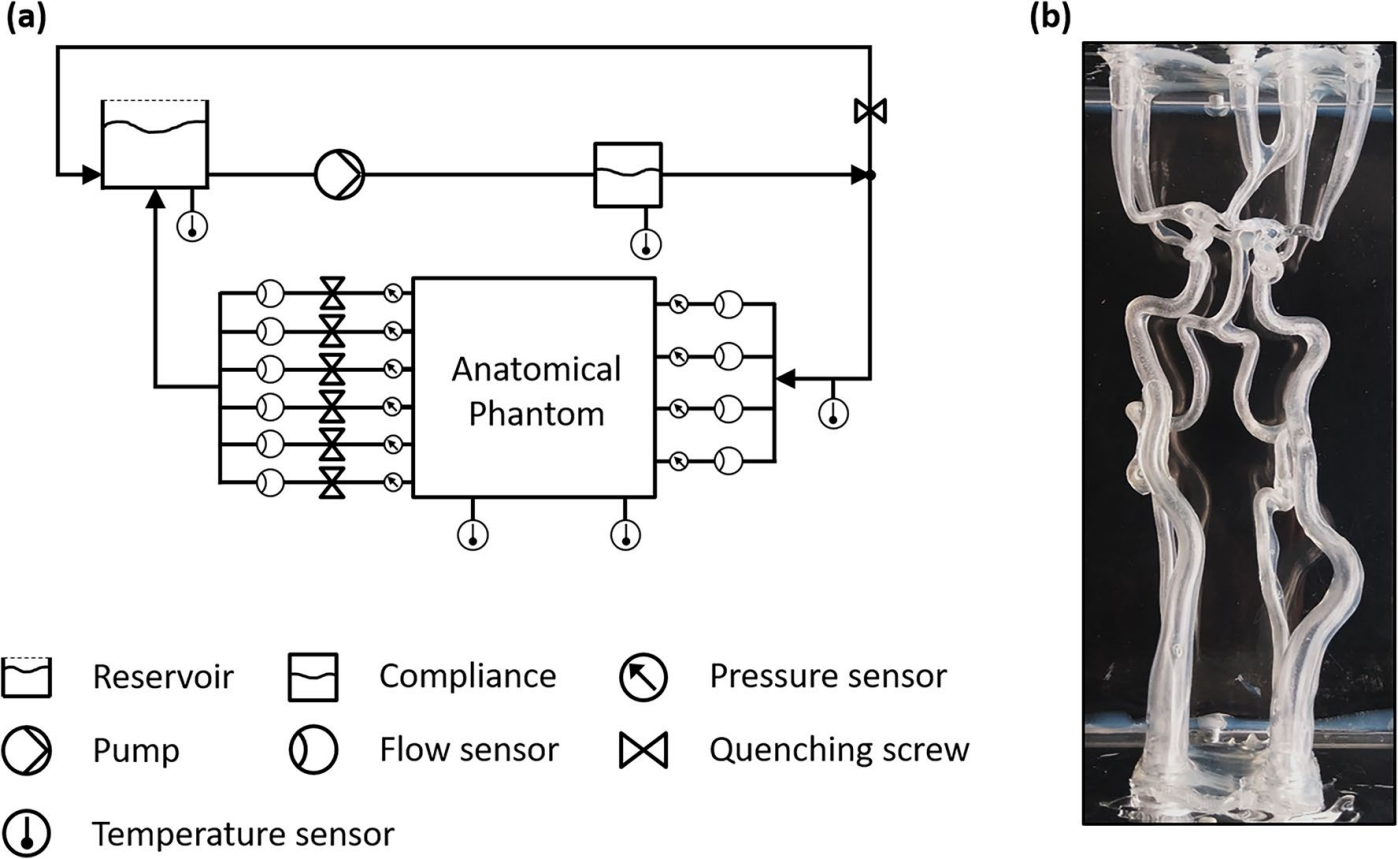
Setup of the in vitro experiment: (**a**) scheme of the setup, (**b**) image of the anatomical phantom.

For the validation of the transient in-silico model, we set up an experiment allowing for the flow investigation on anatomical phantoms of cerebral arteries. Figure 1a illustrates the experimental setup consisting of a closed test loop with an open reservoir. The experiment was set up to reproduce transient hemodynamic parameters of the cerebral circulation observed in-vivo and overcomes several limitations of our previous study^13^ by improving following factors:

- new phantom model without material defects
- vessel diameters averaged from a cohort of 100 patients
- high-resolution flow and pressure data acquisition

A displacement pump was used to generate transient physiological flows and pressures with 40:60% for the ratio of duration of systole to diastole. The pump ran at a frequency of 60 beats per minute (f = 1 Hz). The quenching screw of the bypass was adjusted targeting the physiological total cerebral blood flow (tCBF) and the PVC tubes downstream of the anatomical phantom were quenched in order to adjust the tCBF distribution at the outlets, while the remaining fluid was bypassed into the reservoir. Physiological flow curves at the inlets of the anatomical phantom were achieved by the use of a tunable aortic compliance. During the experiments, time-resolved data of flows and static pressures for all inlets and outlets were recorded simultaneously.

For this study, a phantom model with complete CoW anatomy was created (see Fig. 1b) for the cerebrovascular anatomy from the base of the skull to the A2 segment of the left and right anterior cerebral artery (LACA and RACA), to the M1 segment of the left and right middle cerebral artery (LMCA and RMCA), and to the P2 segment of the left and right posterior cerebral arteries (LPCA and RPCA). We used CTA scans of one random patient with a complete CoW and segment the anatomy with the software Mimics 15.0 to derive the vascular structure centerlines. To create a cohort averaged geometry, we then performed sweeping operations of the averaged vessel diameters from a group of 100 patients along the cross-sections of the centerlines. A virtual healthy anatomy (based on the 100 subjects) was used because the in-vivo data, which was taken for the calibration of the in-vitro model, has also been gathered from a healthy population. Finally, the inlets and outlets of the 3D model were extended perpendicular to the inlet and outlet planes of the box, respectively. All methods were performed in accordance with the relevant guidelines and regulations. For details on the vessel diameters, please refer to the Supplementary Table S1 online. The data handling was approved by the ethics review committee of the University Hospital RWTH Aachen (number of ethics approval: 335–15) and the participants gave informed consent to participate in the study before taking part. A detailed description of the manufacturing process for creating this type of anatomical phantoms was described previously^13^. The wall thickness of the model was approximately 1 mm and Young’s modulus of the model material was approximately 0.53 MPa at normal temperature and pressure, close to results from human MCA postmortem studies (0.42 MPa)^21^ and in agreement with other experimental studies^12^.

A water-glycerol mixture kept at 45 °C ± 1 °C was used as a blood analogue for the test loop with a dynamic viscosity of 4.01 ± 0.02 mPas measured with a cone-plate rheometer and a density of 1.137 g mL^−1^ at the nominal temperature^22^. The anatomical phantom was suspended in the same fluid within an acrylic glass box (210 × 145 × 90 mm^3^ inner dimensions), modeling the surrounding brain tissue of the arteries and having a dampening effect on the oscillations of the phantom during the cardiac cycle.

### Experimental data acquisition

Three complete cardiac cycles were recorded with our data acquisition system ensuring time-resolved flow and pressure data recording for all inlets and outlets simultaneously with a frequency of 1 kHz. 10 pressure sensors with pressure transducers were placed at the four inlets and six outlets of the anatomical phantom, measuring the static pressure. We estimated the uncertainty of the pressure measurement to be ± 0.5 mmHg from the nominal value after calibration. 11 ultrasound flow sensors were calibrated in a separate calibration loop and then placed at the inlets and outlets of the anatomical phantom as well as downstream to the outlet vessels measuring the tCBF. The sensors were placed downstream of straight parts of the PVC tubes to ensure developed flow profiles at the measuring locations in order to reduce measurement inaccuracies. We estimated the uncertainty of the flow measurement to be ± 5 mL min^−1^ from the nominal value. The flow signals were filtered during post processing with a 6th order Butterworth low pass filter with a cutoff frequency of 7 Hz to reduce noise. All data were evaluated and post processed with Matlab R2021b (The Mathworks Inc., Natick, USA). The acquired data is available to the research community in the Supplementary Data (for more details see section Data availability).

### In-silico setup

**Figure 2:**
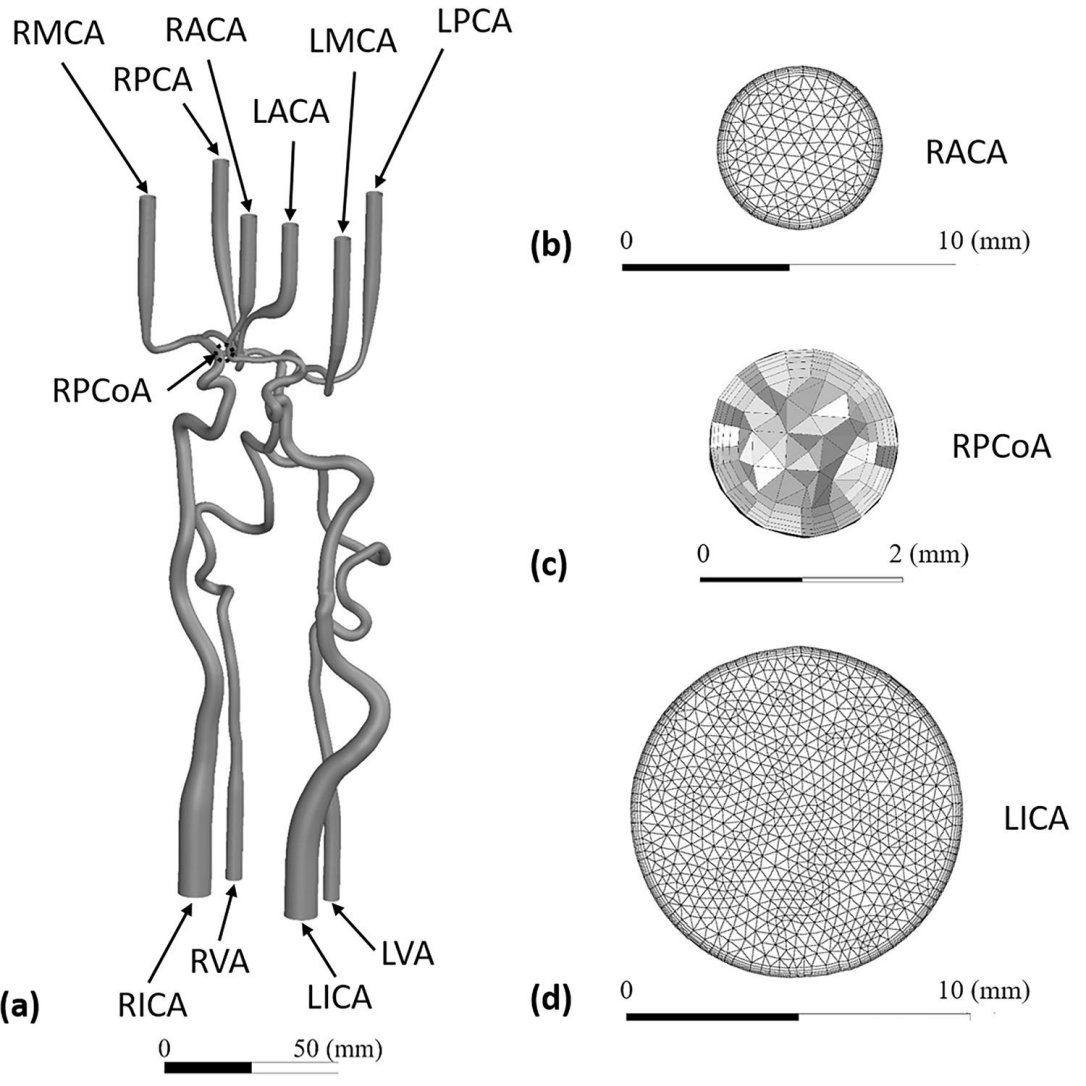
Details of the in-silico setup and computational mesh at characteristic vessel sizes: (**a**) CFD model geometry and naming of the arteries, highlighting the inner right posterior communicating artery (RPCoA) in dotted circle, (**b**) surface mesh at the RACA outlet, (**c**) volume mesh of the RPCoA with small vessel diameter, (**d**) surface mesh at the LICA inlet.

The same model geometry, which was used for producing the anatomical phantom for the experimental analysis, was utilized for the 3D CFD model (see Fig. 2a). The meshing tool Ansys ICEM CFD 2021 R2 (Ansys Inc, Canonburg, PA, USA) was used to generate a mesh for the model geometry. The CFD analysis was conducted via Ansys Fluent 2021 R2, solving conservation equations for mass and momentum with a pressure-based solver. The pressure based solver was used as it was developed specifically for incompressible flows. The governing equations were solved with a segregated algorithm, in which pressure and velocity were coupled via pressure-implicit with splitting of operators (PISO) scheme. Laminar flow was assumed based on the findings of CFD investigations in cerebral arteries^23^. An incompressible fluid with the same fluid properties of the blood analogue from the experiment was used for the numerical analysis; further, a no slip condition at the walls was set. A grid refining study led to the final unstructured mesh consisting of around 3.6 × 10^6^ elements with 5 inflating prism layers near the walls for this study (see Fig. 2 b–d). The transient simulations were conducted with a physical time step of 1 ms for three cardiac cycles in order to eliminate initial variable disturbance and using only the last cardiac cycle for the evaluation. Solution convergence was assumed with absolute convergence criterion for residuals of continuity and velocity below 10^−5^.

### Boundary conditions

All simulations were implemented with transient mass flows at the inlets, derived from the experimental volumetric flows (see Supplementary Fig. S1 online) scaled with the density of the fluid from the experiment. At the outlets, open boundaries were implemented with different pressure-based approaches. The five approaches for modeling the outlet boundaries are presented in the following. All parameters used for the implementation of the outlet BCs can be found in the Supplementary Table S2 online.

1. In the first approach, one stationary static pressure for all outlets was used, referred to as the *zero pressure* (ZP) BC in the literature^15^. This BC is assumed when no detailed information within the intracranial arteries is available; this assumption was made in few studies^15,24^. In this study, one stationary, static pressure of ${{\text{p}}}_{{\text{ZP}}}=\overline{{\text{p}} }=87\mathrm{ mmHg}$ relative to the atmospheric pressure was set for all outlets, determined by cycle-averaging all experimental outlet values.
2. The second approach was a symmetrical description for the left and right side of the cerebrovascular anatomy that was based on a study of stationary cerebral hemodynamics with complete CoW^25^. The outlet pressures
  1 $${p}_{SP}={\overline{p} }_{n}+ \frac{1}{2}{c}_{n}\rho {\left(\frac{Q}{A}\right)}^{2}$$
  for the *symmetrical pressure* (SP) approach were defined according to Eq. (1). Cycle-averaged static pressures at each outlet were derived from the experiment, and after averaging the values from the left and right vessel segments of the same kind, the stationary, relative static pressures ${\overline{{\text{p}}} }_{{\text{n}}}$ were equally implemented on both sides for the anterior (LACA and RACA), media (LMCA and RMCA) and posterior artery (LPCA and RPCA) outlets. Hydraulic loss coefficient values c_n_ were adopted from the reported study^25^, while outlet areas A were given by the anatomical model (see Supplementary Table S3 online). This approach described a quasi-stationary case due to the low volumetric flows Q at the boundaries.
3. In the third approach, transient pressure curves were implemented for each outlet directly from the experimental data. This approach was named direct experimental pressure (DEP) and it was used as a metric for comparison of the other BCs.
4. A coupled lumped-parameter model gave the outlet BCs in the fourth approach. The model consisted of two elements, a capacitance C and a resistance R in parallel, known as Windkessel *model* (WM), with R accounting for the pressure drop ∆p in the peripheral circulation^26^. Resistance values for each vessel were derived by curve fitting the linear relationship in Eq. (2)
  2 $$\Delta p=R\cdot Q$$
  to the transient experimental data. The resistance values for the model were determined by averaging the values from the left and right side of the same kind of vessel (ACA, MCA, and PCA). The MCA capacitance value was estimated from in-vivo data of the normalized compliance of MCA^27^. The capacitance values for the other vessels were determined by the constraint that the characteristic time $\uptau ={\text{R}}\cdot {\text{C}}$ was uniform for all outlets, based on the finding that an increased compliance reduces the vascular resistance in cerebral arteries^27^. The ordinary differential equation of the WM was implemented as a user defined function and solved with an explicit Euler scheme in the same time step as the transient simulation.
5. The idea for the fifth set of BCs was to propose a generalizable boundary condition that mathematically describes transient pressures from the experimental observations of this study. For describing the pressure, phase modulation (PM) method from communication systems was used^28^. We described all six transient outlet pressure curves with the same parameter set and varied only the mean pressure levels for the single outlets. Equation (3)
  3 $${p}_{PM}={\overline{p} }_{n}\cdot \left(1+\frac{{PI}_{p}}{2}\cdot cos\left(2\pi f\cdot t+\varphi +\beta \cdot cos\left(2\pi f\cdot t+\varphi \right)\right)\right)$$
  was used to generate transient pressure functions through a phenomenological description and thus approximation of our experimental data. f is the constant frequency of the displacement pump, φ the phase of the pressure signal and β is the modulation index. PI_p_ describes the pressure pulsatility through the ratio of pulse pressure over mean pressure, in analogy to the flow PI in Eq. (4). The parameters φ, β and PI_p_ were derived by curve fitting Eq. (3) to the experimental pressure data for each outlet vessel (see Supplementary Fig. S2 online) and then taking the median of each parameter set. These parameters were kept constant for all outlet pressure functions. Mean pressure values ${\overline{{\text{p}}} }_{{\text{n}}}$ in Eq. (3) were identical to the SP case. This approach has the advantage of preserving the shape of the physiological pressure curves from the experiment while being generally customizable to different pressure levels and cardiac cycle phases.

BC sets ZP and SP were considered stationary pressure approaches, while DEP, PM, and WM accounted for the transient behavior of the outlet pressure.

### Analysis procedure

First, we calibrated the experiment by comparing tCBF, inlet pressure, tCBF distribution, and flow PI values with in-vivo values from literature. Cycle-averaged data included all datapoints of the third cardiac cycle acquired with 1 kHz. Subsequently, we used the in-vitro results as benchmark for evaluating the accuracy of the in-silico modeling approaches in a direct comparison with the following procedure:

1. Transient inlet pressures were assessed, as they were calculated quantities from the simulations.
2. Percentage distribution of tCBF to the outlets was verified using cycle-averaged outlet flows.
3. Transient outlet flows were evaluated, as they were calculated quantities from the simulations.
4. From the transient outlet flow curves, flow PI values were calculated according to Eq. (4)
  4 $$PI=\frac{{\widehat{Q}}_{systolic}-{\widehat{Q}}_{diastolic}}{\overline{Q} }$$
  as the quotient of the flow amplitude between the peak-systolic flow ${\widehat{{\text{Q}}}}_{{\text{systolic}}}$ and end-diastolic flow ${\widehat{{\text{Q}}}}_{{\text{diastolic}}}$ over the mean flow $\overline{{\text{Q}} }$ ^4^.
5. Time-dependent deviation between the numerical and experimental data was quantified using root mean squared deviation (RMSD) values of the outlet pressures, inlet pressures, and outlet flows. Subsequently, normalized root mean squared deviation (NRMSD) values were derived by normalizing the RMSD values to the cycle-averaged experimental values.

## Results

**Figure 3:**
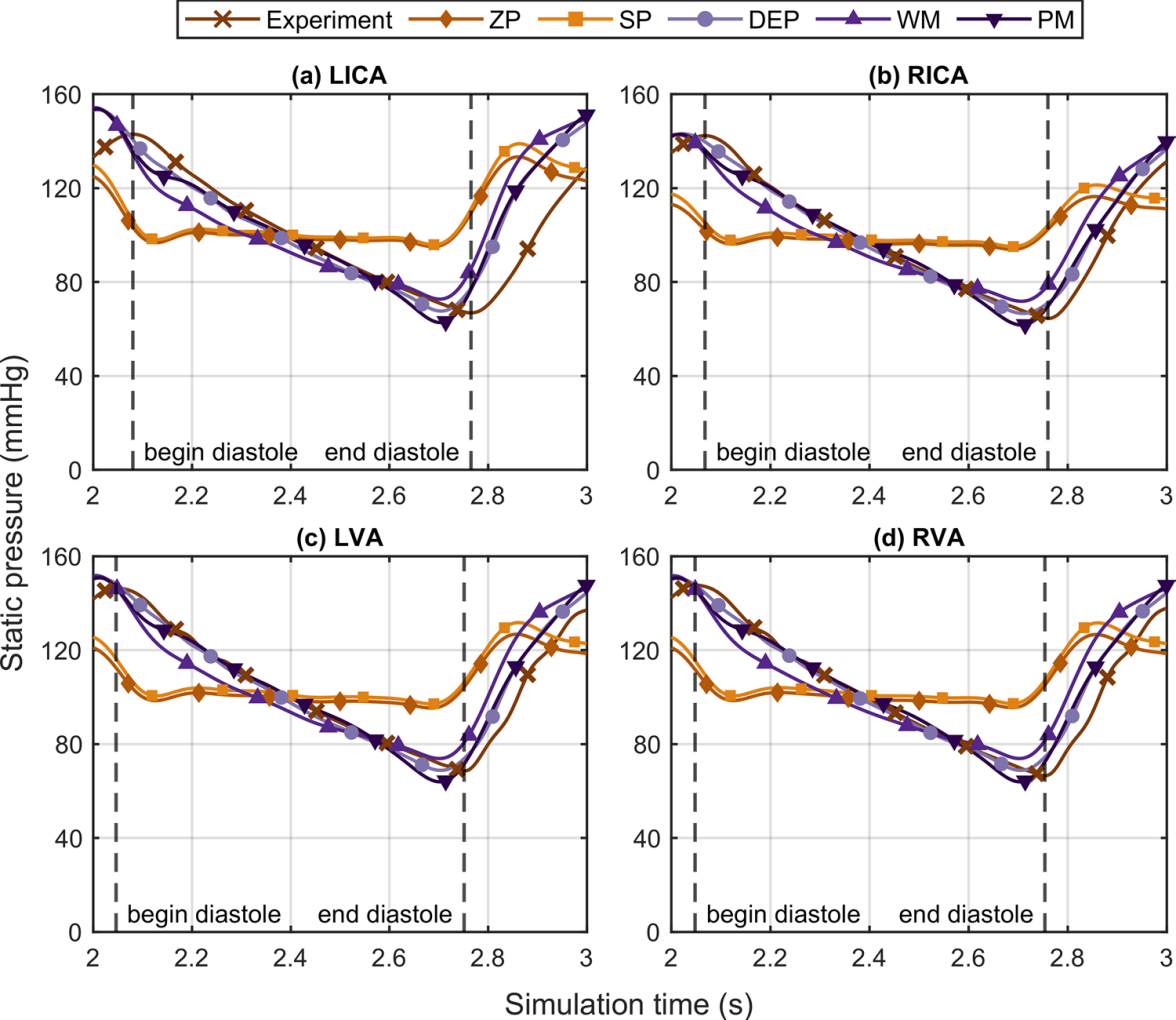
Comparison of the transient pressures at the four inlet vessels: (**a**) LICA, (**b**) RICA, (**c**) LVA, and (**d**) RVA. For each inlet vessel, pressure curves from the experiment and the five outlet boundary condition sets are plotted. *ZP* zero pressure, *SP* symmetrical pressure, *DEP* direct experimental pressure, *WM* Windkessel model, *PM* phase modulation. For the experiment, begin and end of the diastolic phase are depicted with dashed vertical lines for each inlet artery.

We compared our experimental results to in-vivo measurements of similar tCBF regarding flow distribution and flow PI values. Inlet flows were compared to the studies of Zarrinkoob et al.^5,6^, while the outlets were compared to the study of Correia de Verdier and Wikström^4^. The tCBF and distribution of tCBF flow in Table 1 indicates that experimental values were properly set to reported in-vivo values. Additionally, flow PI values correspond to the in-vivo measurements, except for the LICA flow PI value. Moreover, cycle-averaged inlet pressures ranged from 101 to 105 mmHg and inlet pulse pressure from 76 to 81 mmHg (see Fig. 3), in consistency with the comparing study^6^. Cycle-averaged outlet pressures ranged from 84 to 91 mmHg and outlet pulse pressure from 66 to 73 mmHg (see Supplementary Fig. S3 online). The mean pressure decreased from inlets to outlets on average by 16% while the mean pulse pressure decreased on average by 10%. In the following, details of the experimental results are presented with the in-silico results for a direct comparison.

Transient inlet pressures are shown in Fig. 3. As reference, dashed vertical lines in Fig. 3 mark begin and the end of the diastolic phase for the experiment. The experimental results indicate that the pressure phases are different for the ICA and the VA, but similar between the left and right side. Compared to the experiment, all simulations resulted in a pressure phase that is earlier in time and corresponds to the phase of the imposed inlet flows (see Supplementary Fig. S1 online). Moreover, transient cases overestimated the inlet pressures at the beginning of the diastole, up to a difference of 10 mmHg at the LICA. The end-diastolic pressure of the experiment was best captured by the DEP approach, while the PM approach underestimated the end-diastolic pressure and the WM overestimated it. Only marginal differences were found for the inlet pressure curves between the two stationary pressure approaches ZP and SP, which both gave a pulse pressure of less than half of the experimental values. Cycle-averaged inlet pressures of the simulations deviated less than 5% from the experimental values.

**Figure 4:**
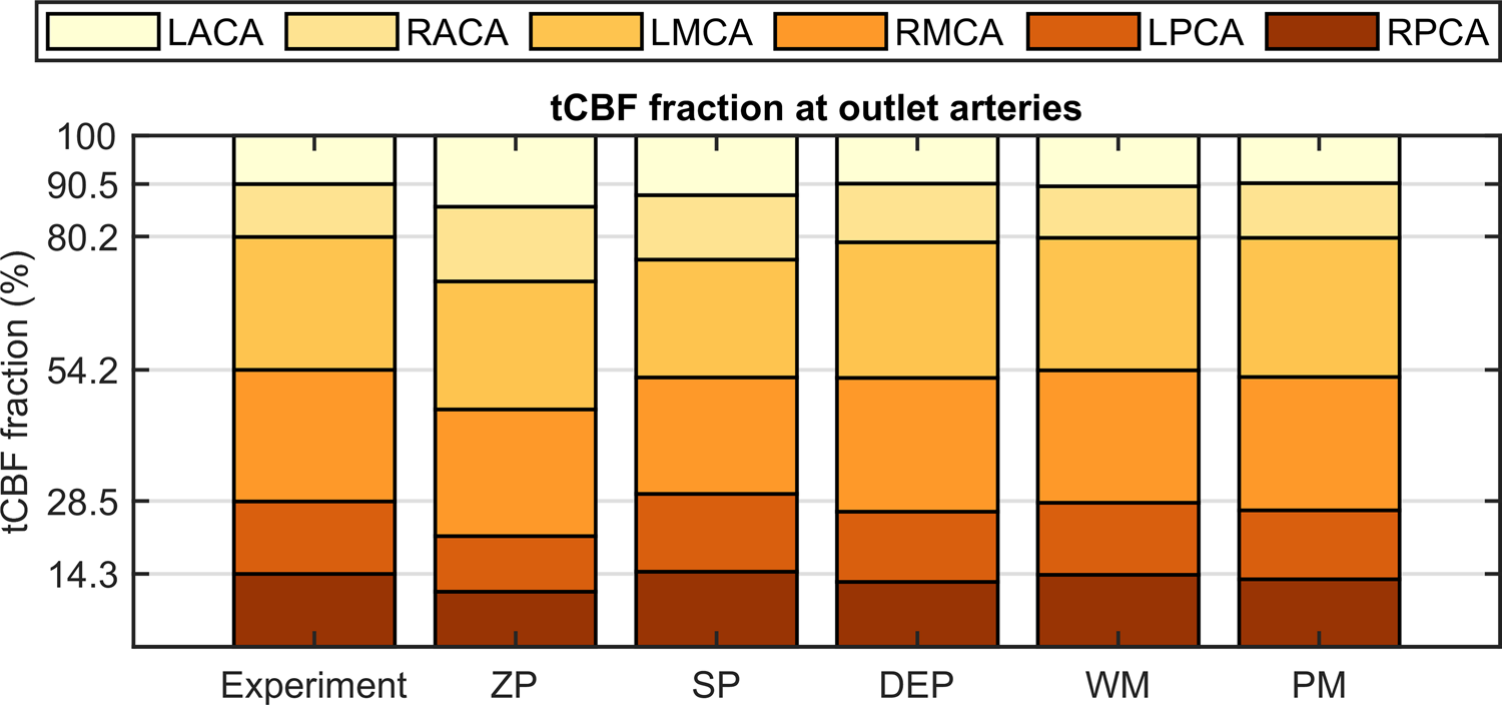
Percentage distribution of tCBF to the outlet vessels RPCA, LPCA, RMCA, LMCA, RACA, and LACA for the experiment and the five outlet boundary condition sets. *ZP* zero pressure, *SP* symmetrical pressure, *DEP* direct experimental pressure, *WM* Windkessel model, *PM* phase modulation. Dashed lines mark the timing of experimental end diastolic flow.

The cycle-averaged outlet flow distributions in tCBF are illustrated in Fig. 4 and show that the WM best captured the experimental flow distribution. The tCBF fractions showed minor differences to the experimental results for the DEP, and PM approach, but higher ones for the SP and ZP approach. In the latter case, there was a shift of flow from the anterior to the posterior circulation due to the pressure inconsistency compared to the experimental results. Cycle-averaged outlet flows of the simulations deviated 1–45% from the experimental values.

**Figure 5:**
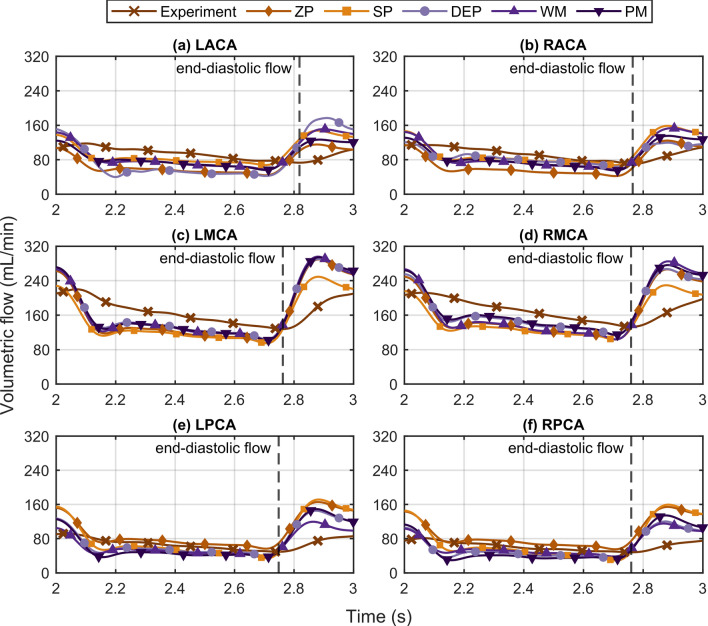
Comparison of the transient flows at the six outlet vessels: (**a**) LACA, (**b**) RACA, (**c**) LMCA, (**d**) RMCA, (**e**) LPCA, and (**f**) RPCA. For each outlet vessel, flow curves from the experiment and the five outlet boundary condition sets are plotted. *ZP* zero pressure, *SP* symmetrical pressure, *DEP* direct experimental pressure, *WM* Windkessel model, *PM* phase modulation.

Transient outflow curves are depicted in Fig. 5 with dashed lines marking the end-diastolic flow of the experimental curves. The experimental results indicate that the end-diastolic flow phases are slightly shifted from the anterior (ACA) to middle (MCA), and to posterior (PCA) cerebral arteries towards earlier times. In comparison to the inlet flow curves (see Supplementary Fig. S1 online), a time-shift in the experimental flow curves was observed from the inlets to the outlets. On the contrary, there was no time-shift in the flow curves of the in-silico model. Moreover, the flow amplitudes given by the simulations were throughout higher than for the experimental values. Particularly, the flow amplitude of the LACA curve was found to be the highest for the DEP approach (Fig. 5a), although the cycle-averaged LACA flow was in line with the experimental results (see Fig. 4).

**Figure 6:**
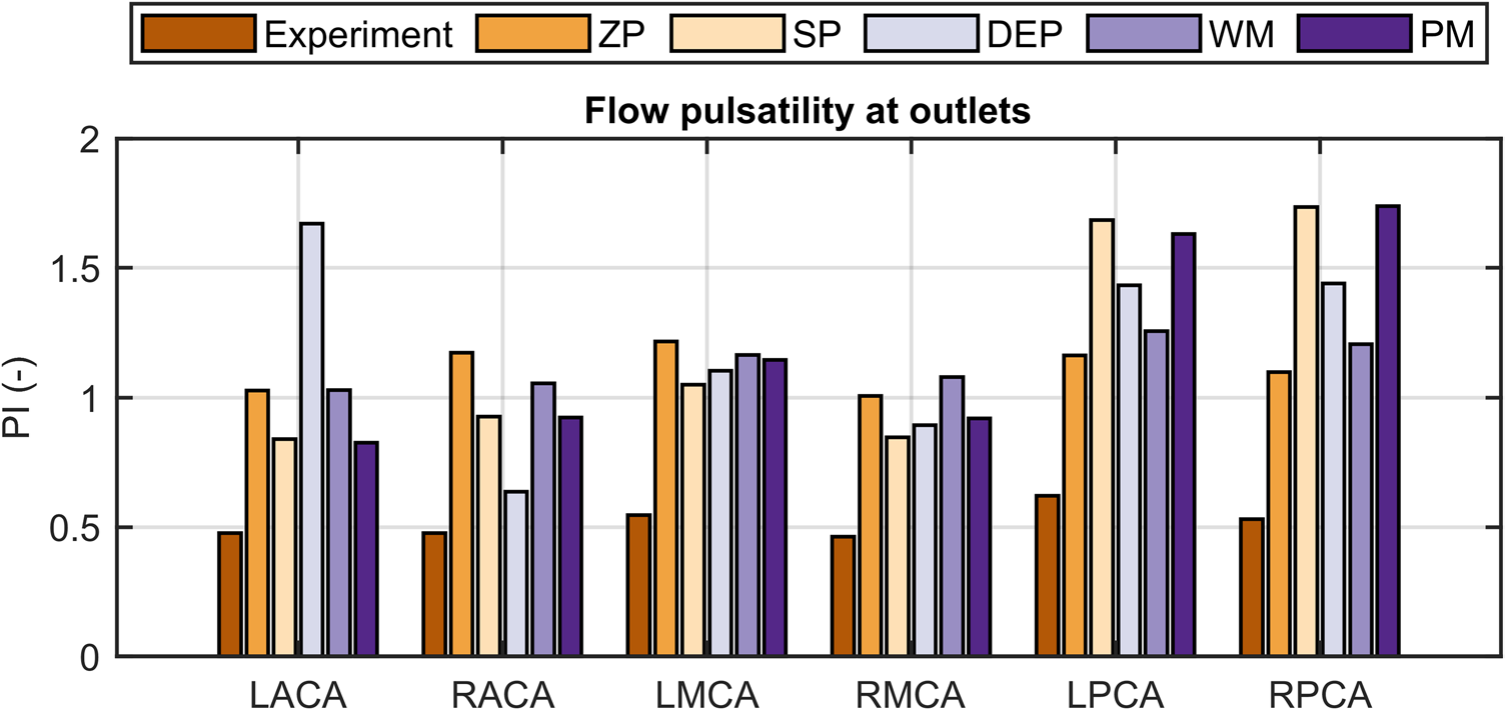
Flow PI values at the six outlet vessels (LACA, RACA, LMCA, RMCA, LPCA, and RPCA) for the experiment and for the five outlet boundary condition sets. *ZP* zero pressure, *SP* symmetrical pressure, *DEP* direct experimental pressure, *WM* Windkessel model, *PM* phase modulation.

Outlet flow PI values are illustrated in Fig. 6. In comparison to the experimental findings, the in-silico model overestimated the flow pulsatility for all BC sets. Flow PI values of the in-silico model exceeded the in-vitro results in particular at both left and right PCA (LPCA and RPCA) and at the LACA artery for the DEP approach. On the other hand, both WM and ZP approaches led to evenly distributed flow PI values at the outlets although values were approximately twice as high as experimentally determined.

**Figure 7:**
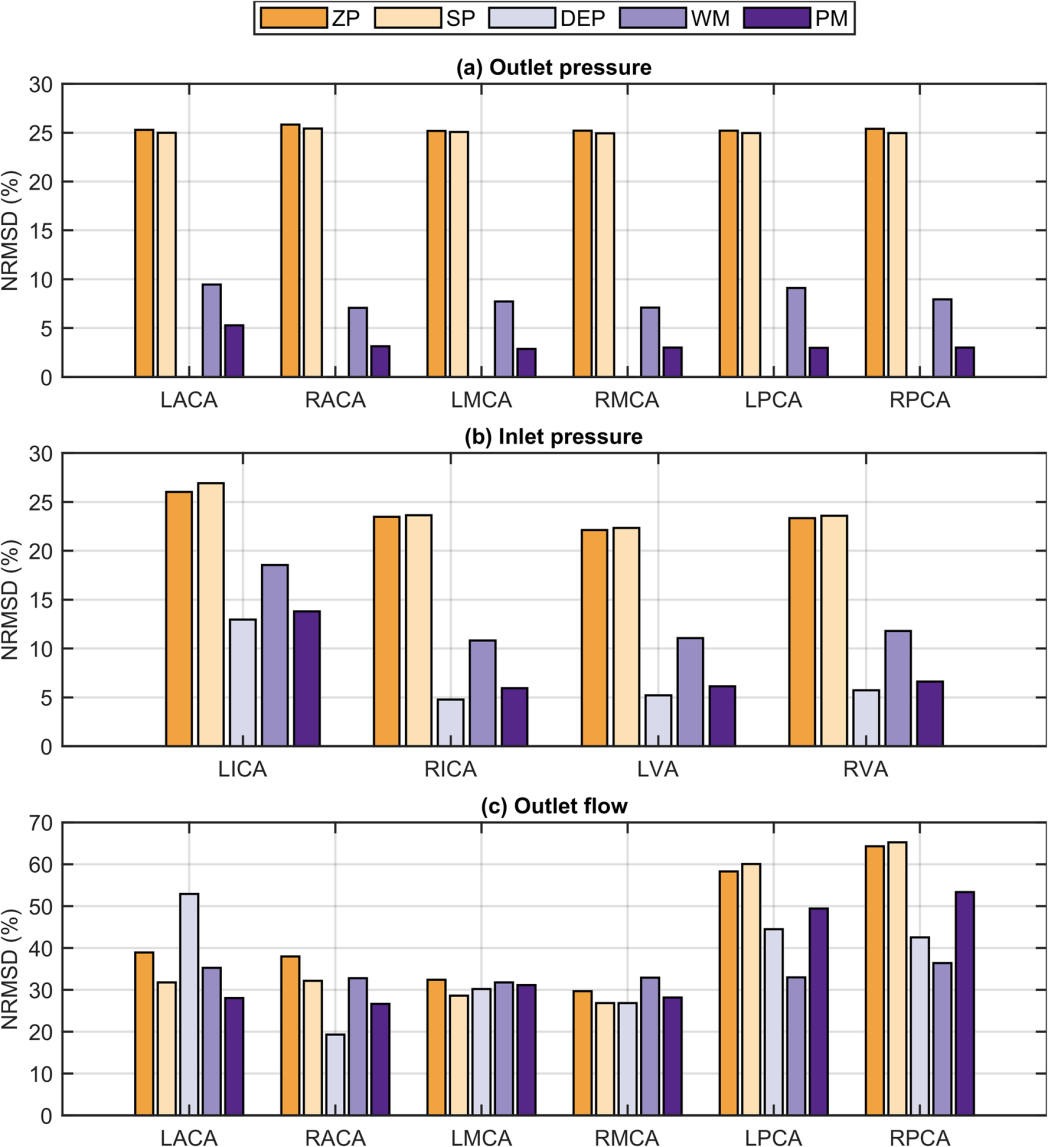
NRMSD values of all outlet boundary condition sets for (**a**) outlet pressure, (**b**) inlet pressure, and (**c**) outlet flow at the six outlet vessels (LACA, RACA, LMCA, RMCA, LPCA, and RPCA) and four inlet vessels (LICA, RICA, LVA, RVA). *ZP* zero pressure, *SP* symmetrical pressure, *DEP* direct experimental pressure, *WM* Windkessel model, *PM* phase modulation.

Time-dependent deviations between the modeling approaches and the experiment are represented by the NRMSD, whose values are illustrated in Fig. 7. The deviations are subdivided into outlet pressure (Fig. 7a), inlet pressure (Fig. 7b) and outlet flow (Fig. 7c). Outlet pressure NRMSD (Fig. 7a) for both stationary pressure approaches reached values of 25–26%. The DEP approach replicated the experimental outlet pressure values (NRMSD < 0.002%). In contrast, the transient approximations and generalizations of the experimental outlet pressure BCs led to NRMSD < 10% for the WM and NRMSD < 6% for the PM approach. Inlet pressure NRMSD values (Fig. 7b) show a qualitative agreement the outlet pressure NRMSD values (Fig. 7a) for all BC sets. Specifically, both stationary pressure approaches resulted in the highest inlet pressure NRMSD values of 22–27%. While the WM led to inlet pressure NRMSD values of 11–19%, the PM approach reached inlet pressure NRMSD values of 6–14% being just slightly higher than the DEP approach NRMSD values of 5–13%. Inlet pressure NRMSD values were overall higher for the LICA than for the other three inlets that resulted in similar deviations for the different modeling approaches. In terms of outlet flow NRMSD (Fig. 7c), generally higher values were reached compared to the pressure NRMSD values, particularly at the posterior arteries (LPCA and RPCA). The SP approach led to the highest outlet flow deviations (NRMSD values of 27–65%), while the ZP approach showed NRMSD values of 30–64%. Surprisingly, the DEP approach resulted in outlet flow NRMSD values of 19–53% due to a large deviation of the LACA flow. This large deviation is due to the pronounced flow amplitude depicted Fig. 5a. The WM led to outlet flow NRMSD values of 32–36%, which interestingly span the smallest range among all approaches. The PM approach led to outlet flow NRMSD values of 27–53%.

## Discussion

The objective of this study was to evaluate cerebrovascular CFD modeling accuracy and identify the needed modeling detail for the study of cerebral hemodynamics. To achieve this objective, we analyzed transient cerebral hemodynamics in a combined in-silico and in-vitro study on a phantom model of the same cerebrovascular anatomy calibrated to in-vivo data. Evaluating the CFD modeling accuracy included assessing physiological flow properties of the intracranial outlet flows, such as percentage distribution of tCBF and flow PI values, as well as quantifying time-dependent NRMSD values of the cerebrovascular pressures and flows.

First, we conducted an in-vitro experiment using an anatomical phantom with complete CoW, for which we determined both time-resolved flows and pressures at the inlets and outlets. Then, we used the experimental flows as inlet conditions for the in-silico model that shared the geometry of the anatomical phantom. During the in-silico investigation, we systematically analyzed the effect of various pressure-based outlet BCs with increasing modeling complexity, ranging from reported approaches to new ones, on transient cerebral hemodynamic properties. By comparing the hemodynamic results of the different sets of outlet BCs (ZP, SP, DEP, WM, and PM) with the time-resolved in-vitro results, we draw conclusions for defining the intracranial outlets.

The in-vitro experiment is considered ground truth for the transient analysis for the following reasons. The experimental inlet pressures align with physiological pressures and correspond to pressure amplitudes determined in-vivo^6^. Furthermore, both experimentally obtained tCBF distribution and flow PI values are consistent with reported in-vivo data^4–6^. Moreover, the compliant model has a physiological Young’s modulus^21^ and the phantom’s complete CoW geometry is derived from clinical data. In conclusion, we are confident that our experiment replicates a physiological and clinically plausible case, expanding existing in-vitro setups for investigating cerebral hemodynamics^12,13,23^. Finally, the presented experimental method, characterized by physiological hemodynamics and high-resolution measurements of cerebrovascular flows and pressures, enables a comprehensive validation and evaluation of the modeling accuracy of cerebral hemodynamics.

Flow distribution in the intracranial arteries is clinically related to cerebral perfusion^19^. Regarding the in-silico part of the study, cycle-averaged outlet flow distribution (see Fig. 4) is compromised in the ZP approach, commonly referred to as the zero-pressure case, while the flow distribution remains accurate for the other modeling approaches. In summary, realistic outlet flow distribution can be obtained by considering the hydrostatic pressure difference between the intracranial outlets, either explicitly (SP, DEP, and PM approaches) or implicitly (WM). Our results confirm the findings of previous studies^15,16^, that the ZP approach should be avoided when conducting in-silico investigations of cerebral hemodynamics in order to avoid erroneous results.

Transient inlet pressures are strongly affected by pressure-based outlet BCs with given inflow conditions, as in case of patient-specific models^19^. The transient boundary condition sets (DEP, WM, PM) generate physiological inlet pressure amplitudes (see Fig. 3) and moderate deviations < 19% from the experimental values (see Fig. 7b), whereas the stationary boundary condition sets (ZP and SP) systematically underestimate the inlet pressure amplitudes, resulting in higher transient inlet pressure deviations. When analyzing EVT, such as endovascular aspiration and thrombectomy, it is most relevant to accurately resolve the transient arterial pressure for two reasons. First, the pressure difference from the artery to the aspiration catheter tip alters the aspiration flow^29^, and second, cerebral artery pressure substantially influences the force required during endovascular thrombectomy^11^. In conclusion, stationary outlet BCs should be avoided when modeling transient hemodynamics in the intracranial arteries. On the contrary, the PM approach more accurately resolves the time-dependent arterial pressure due to the moderate inlet pressure NRMSD values < 14%. For this reason, the PM approach holds promise as an alternative boundary condition for transient cerebral hemodynamic investigations and for estimating physiological outlet pressure BCs for the intracranial arteries due to its generalizable form. While the constant pressure pulsatility PI_p_ can be estimated from clinical pressure measurements of the mean aortic pressure, the phase of the pressure signal φ is adapted to the cardiac cycle phase. The experimental results indicate for the presented anatomy that both mean pressure and pulse pressure decreased from inlets to outlets by 16% and 10%, respectively.

Physiological cerebral flow within the intracranial arteries is pulsatile and characterized by the flow PI^4,6^. Although cycle-averaged flow distributions mostly agree to the in-vitro results (see Fig. 4), the in-silico model overestimates the outlet flow pulsatility (see Fig. 6) because of the rigid-wall assumption. Notably, the majority of the in-silico PI values align with the broad range of reported in-vivo measurements listed in Table 1, except for the posterior arteries and for the LACA in case of the DEP approach in particular. However, the flow pulsatility is strongly affected by the different modeling approaches. Both WM and ZP approaches led to evenly distributed flow PI values at the outlets, consistent with the uniformity of average flow PI values of the in-vitro and reported in-vivo results^4,6^. Interestingly, only the WM simultaneously accounts for accurate mean outlet flows, which are indispensable for correct assessment of the flow PI values as seen in Eq. (4). Resistance parameters of the WM are similar to a comparable study^19^, while the capacitance values in this study are lower. Although the WM is considered the most realistic approach for resolving flow pulsatility, outlet flow NRMSD values were in the range 32–36% (see Fig. 7c), while inlet pressure NRMSD values reached 11–19% with this approach (see Fig. 7b).

**Table 1:** Results from the experimental part of this study and comparing literature.

	Experiment			Zarrinkoob et al. ^5,6^		
	Flow rate ± s.d (mL min^−1^)	tCBF fraction (%)	PI ± s.d (–)	Flow rate ± s.d (mL min^−1^)	tCBF fraction (%)	PI ± s.d (–)
tCBF in	659.1	100	–	657 ± 94	100	–
LICA	249.5 ± 2.7	38	1.21 ± 0.05	236 ± 41	36	0.96 ± 0.15
RICA	241.0 ± 2.0	37	0.89 ± 0.03	236 ± 41	36	0.96 ± 0.15
LVA	87.8 ± 1.1	13	1.08 ± 0.07	90 ± 17	14	1.11 ± 0.18
RVA	80.9 ± 1.4	12	1.07 ± 0.06	90 ± 17	14	1.11 ± 0.18

				Correia de Verdier and Wikström^4^		
				Flow rate (range) (mL min^−1^)	tCBF fraction (%)	PI (range) (–)
tCBF out	660.1	100	–	698	100	–
LACA	94.2 ± 0.8	14	0.45 ± 0.02	93 (28–195)	13	0.60 (0.18–1.57)
RACA	93.8 ± 0.7	14	0.50 ± 0.03	113 (36–190)	16	0.67 (0.23–1.19)
LMCA	170.1 ± 1.3	26	0.53 ± 0.02	169 (111–255)	24	0.71 (0.38–1.54)
RMCA	171.8 ± 0.8	26	0.46 ± 0.01	174 (127–264)	25	0.69 (0.44–1.64)
LPCA	68.7 ± 0.7	10	0.60 ± 0.02	77 (31–133)	11	0.58 (0.26–1.00)
RPCA	62.4 ± 0.5	9	0.52 ± 0.01	72 (22–115)	10	0.56 (0.16–0.86)

Cycle-averaged experimental results for all cycles are listed on the left side: mean flow rates, flow distribution as tCBF fraction and flow pulsatility indices at the corresponding arteries. Next to our data, in-vivo data from MRI-based measurements of flow rate and pulsatility indices^4–6^. The values in the column for tCBF fraction of the literature values were calculated based on both the reported mean flow rates and tCBF and were not comprised in the reported literature. For the study of Correia de Verdier and Wikström, tCBF out value was calculated as the sum of the reported flow rates and was not comprised in the literature.

The consistently high outlet flow NRMSD values can be attributed to the disparity of compliance between the anatomical phantom used in the in-vitro experiments and the rigid-wall CFD model. Rigid-wall CFD models cannot reproduce the dampened amplitudes of the flow at the outlets nor the time-shift of flow curves from inlets to outlets. Our study design intentionally considered this discrepancy in order to evaluate the accuracy of cerebrovascular CFD models, which are predominantly used for the in-silico investigation of cerebral hemodynamics^15,17–20^. Noteworthy, the flow PI values we obtained in silico are in line with the in-silico results obtained with a rigid-wall CFD model for a patient-specific case using Windkessel models at the outlets^19^; however, our experimentally generated physiological case gives considerably lower flow PI values at the outlets (see Fig. 6).

We identified that rigid-wall CFD models of the cerebrovascular system have limited transient accuracy, particularly predicting the outlet flows. In order to increase the accuracy of cerebrovascular in-silico modeling, we propose to implement fluid structure interaction (FSI) within the entire cerebrovascular anatomy. FSI modeling has the potential to replicate the compliant effects on cerebral hemodynamics that are characteristic for native cerebral vessels^6,27^ and were observed experimentally in this study. This advanced modeling approach with potentially higher accuracy, however, incurs substantially higher computational costs.

### Limitations

In this work, WM and PM parameter values were derived from the results of the in-vitro part of this study with the presented anatomical geometry of a cohort of 100 patients. Although derived WM parameters are similar to a comparable clinical study^19^, the application of the parameter values of this study is strictly limited to physiological conditions of a virtual healthy subject since no clinical hemodynamic data was used. The results obtained in this study are based on one physiological state and thus application to in-vivo cases is limited. A sensitivity analysis has to show whether these results can be transferred to different cerebrovascular anatomies, tCBF, heart rates, and pulse pressures. Varying pulse pressure as well as heart rates determine the magnitude of acceleration and deceleration of the transient flows, potentially generating different peak velocities inside the CoW. For future sensitivity studies, these parameters may have an impact on the optimization of both the cell sizes and the time step of a robust CFD model. Moreover, WM and PM parameter values should be verified with different datasets to increase the reliability. While clinical data exclusively provides velocity-derived flows in the cerebrovascular system^19^, this study analyzes both flows and pressures simultaneously for determining time-dependent deviation thereof, presenting a comprehensive evaluation of the CFD modeling accuracy in comparison to an in-vitro experiment. However, nominal fluid properties were implemented for the simulations, while viscosity and density of the blood analogue could vary within the temperature range of the experiments. The uncertainty of the actual fluid properties within this temperature range was less than 3% and consequently the potential random error is considered low for this study. The experimentally determined end-diastolic flow peak at the LACA vessel appears shifted forward in time (see Fig. 5a), presumably explaining the mismatch of flow PI values in the anterior circulation for the DEP approach (see Fig. 6) resulting also in higher NRMSD values (see Fig. 7) for the same BC set. However, since the WM and PM approaches model the outlet pressures, this time-shift is compensated. Major flow rate discrepancies between the numerical and the experimental results were observed at the posterior arteries (see Fig. 7c), where flow rates were at their lowest as seen in Table 1 and Fig. 5. These flow conditions cause approximately 8% of the deviations due to the accuracy of the experimental flow measurements.

## Conclusions

The objective of this study was to evaluate cerebrovascular CFD modeling accuracy and identify the needed modeling detail for the study of cerebral hemodynamics. This study provided a comprehensive analysis of transient cerebral hemodynamic characteristics through validation with high-resolution experimental pressure and flow data. On the basis of our findings, we provide the following indications for defining the intracranial outlets:

1. The zero-pressure boundary condition should be avoided because it leads to a distorted outlet flow distribution.
2. Stationary outlet boundary conditions are not suitable for modeling transient cerebral hemodynamics, as they lead to high inaccuracies in pressure.
3. For the analysis of transient cerebral hemodynamics, both WM and PM approaches are recommended. The WM generates realistic flow pulsatilities, while the PM approach ensures accurate arterial pressures.

Moreover, the results of this study aid in understanding the limitations of the accuracy of current cerebrovascular CFD models. We encourage performing FSI analyses of the entire cerebrovascular system in order to increase the modeling accuracy of cerebrovascular pressures and flows simultaneously that is essential for a detailed study of local cerebral hemodynamics. Thereby refined modeling will enhance the significance and prediction accuracy of cerebrovascular in-silico studies on the pathophysiology of stroke, and may ultimately improve EVT techniques such as aspiration and thrombectomy.

### Supplementary Information

Supplementary Information 1.

Supplementary Information 2.

Supplementary Information 3.

## Data Availability

The complete dataset supporting the conclusions of this article is included within the article and its supplementary files. The Supplementary Information contains graphical information and tabular data supplementary to the methods and results. Dataset 1 contains the time-resolved experimental results for volumetric flows (index q, unit mL min^−1^) and static pressures (index p, unit mmHg) at the four inlets (LICA, RICA, LVA, RVA) and the six outlets (LACA, RACA, LMCA, RMCA, LPCA, RPCA) as CSV-file. Dataset 2 contains the cerebrovascular anatomical model as STL-file.
