# Supplementary material for: Evaluating the accuracy of cerebrovascular computational fluid dynamics modeling through time-resolved experimental validation
Source: Sci Rep. 2024 Apr 8;14:8194. doi: 10.1038/s41598-024-58925-8 (PMC11001858; doi:10.1038/s41598-024-58925-8)
Supplement: Supplementary file 1 — Supplementary Information 1. [file 41598_2024_58925_MOESM1_ESM.pdf]

Supplementary Information

Title

Evaluating the accuracy of cerebrovascular computational fluid dynamics modeling through time-resolved experimental validation

Authors

C. A. Luisi[1]; T. L. Witter[1]; O. Nikoubashman[2]; M. Wiesmann[2]; U. Steinseifer[1], M. Neidlin[1]

[1] Department of Cardiovascular Engineering, Institute of Applied Medical Engineering, Medical Faculty, RWTH Aachen University, Pauwelsstr. 20, 52074 Aachen, Germany

[2] Clinic for Diagnostic and Interventional Neuroradiology, Medical Faculty, RWTH Aachen University, Pauwelsstr. 30, 52074 Aachen, Germany

Corresponding Author

Michael Neidlin

neidlin@ame.rwth-aachen.de

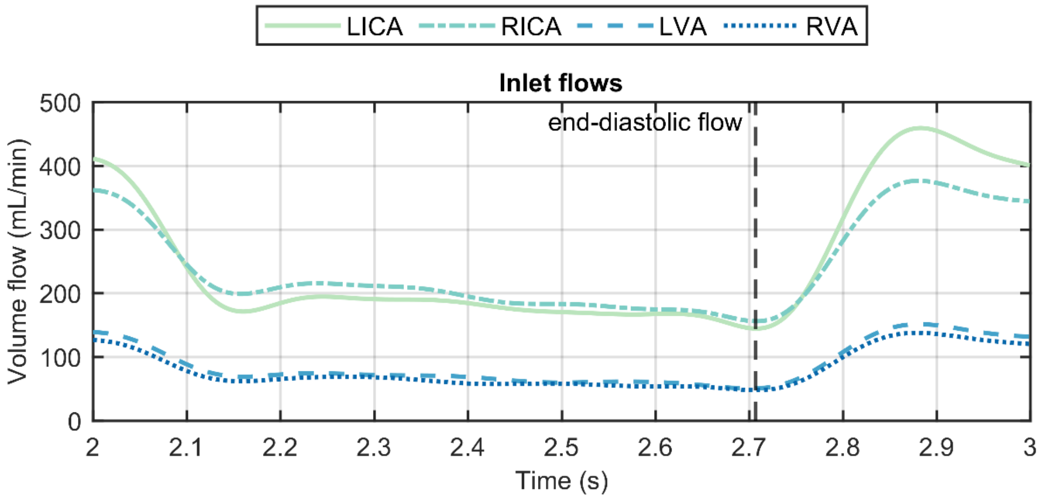

Supplementary Fig. S1 Experimental volumetric flow curves at the four inlet vessels (LICA, RICA, LVA, and RVA) used for defining the inflow conditions for the CFD model. The dashed line marks the timing of the end diastolic flow

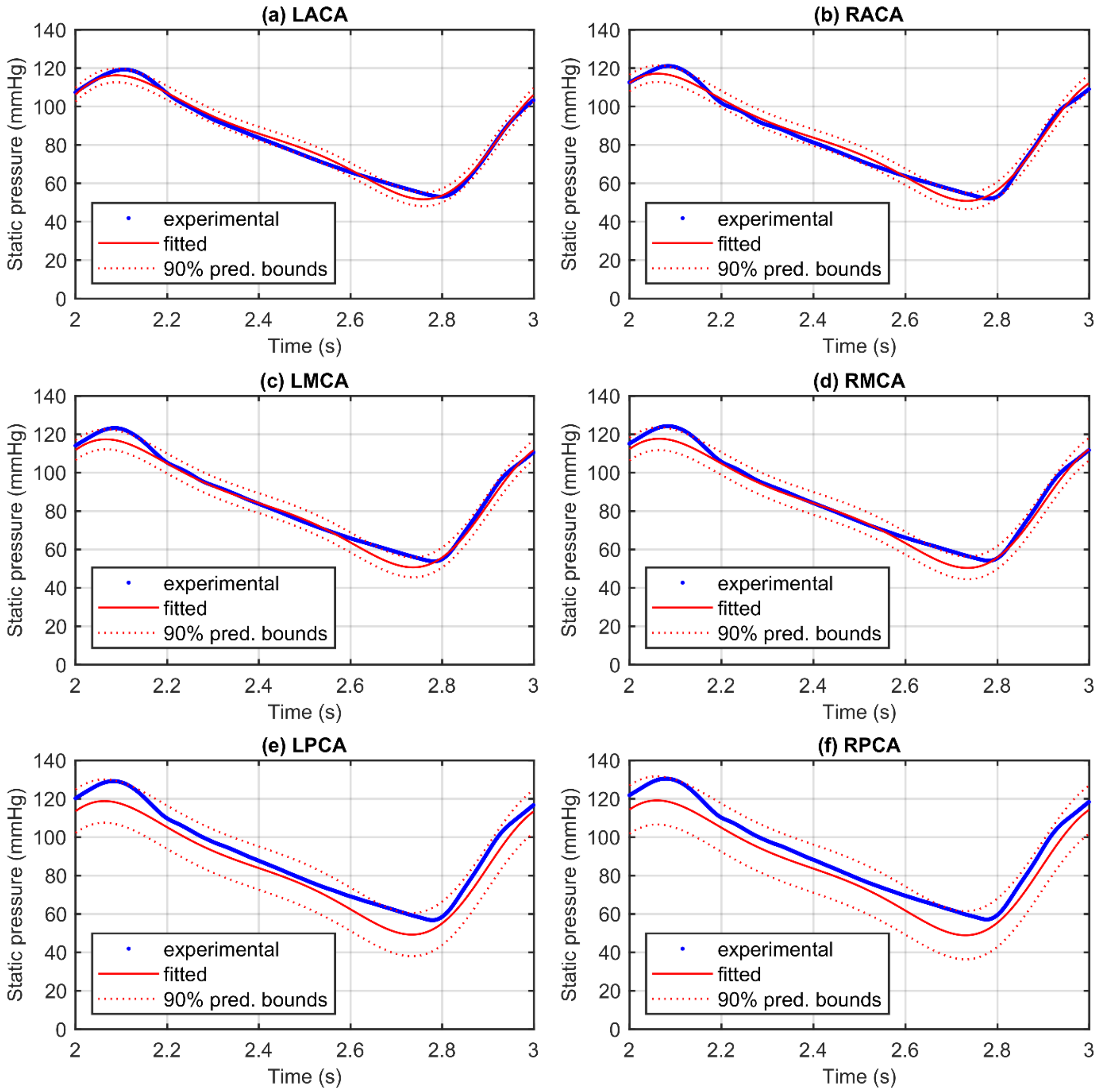

Supplementary Fig. S2 Curve fitting the experimental pressure in order to determine the final parameters φ, β and PI_p of the phase modulation (PM) approach (see Supplementary Table S1)

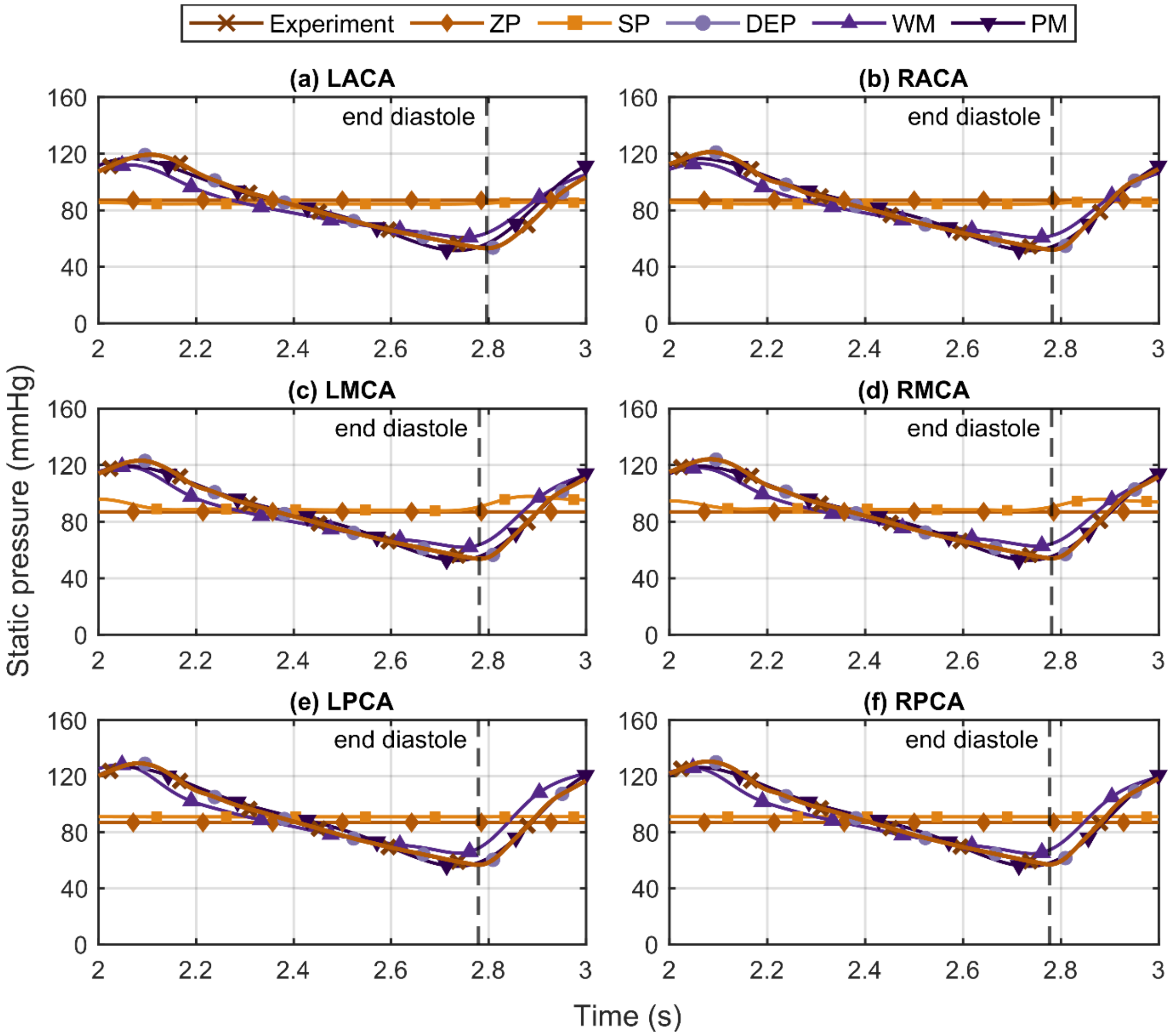

Supplementary Fig. S3 Implemented outlet pressure curves at the six outlets: a) LACA, b) RACA, c) LMCA, d) RMCA, e) LPCA, and f) RPCA. For each inlet vessel, pressure curves from the experiment and the five outlet boundary condition sets are plotted. ZP: zero pressure, SP: symmetrical pressure, DEP: direct experimental pressure, WM: Windkessel model, PM: phase modulation. For the experiment, end of the diastolic phase is depicted with dashed vertical lines for each outlet artery

Supplementary Table S1 Length of the different branches and the mean and s.d. values of the branches diameters measured on the cohort. ICA: internal carotid artery, MCA: middle cerebral artery, ACA: anterior cerebral artery, VA: vertebral artery, PCA: posterior cerebral artery, PCoA: posterior communicating artery, BA: basilar artery.

	**Right**		**Left**	
Vessel name	Diameter	Length	Diameter	Length
	Mean ± s.d. (mm)	Mean (mm)	Mean ± s.d. (mm)	Mean (mm)
ICA origin	6.84±1.72	167.5	6.97±1.63	163.4
ICA skull base	4.48±0.91		4.23±0.82	
ICA ascending siphon	3.71±0.70		3.69±0.57	
ICA terminal segment	3.02±0.56		2.79±0.63	
MCA beginning	2.37±0.37	20.5	2.22±0.43	22.1
MCA end	2.18±0.36		2.10±0.31	
ACA beginning	2.00±0.41	20.0	1.96±0.42	20.5
ACA end	1.87±0.32		1.81±0.35	
VA beginning	3.34±0.74	78.0	3.51±0.68	75.8
VA atlantic segment	3.29±0.72		3.36±0.68	
VA intracranial	2.87±0.76		3.02±0.72	
VA terminal	2.26±0.60		2.41±0.64	
PCA beginning	2.03±0.42	36.1	2.02±0.37	33.1
PCA end	1.85±0.35		1.82±0.37	
PCoA	1.84±1.12	16.2	1.61±0.32	10.1
	No belonging to either right or left hemisphere			
Vessel name	Mean diameter ± s.d. (mm)		Mean length (mm)	
BA beginning	3.05±0.66		31.8	
BA end	2.98±0.59			

Supplementary Table S2 Listing of the outlet boundary condition sets used in the in-silico part of the study. The five outlet boundary condition sets are presented with their full name and abbreviation. It is specified which parameters are used for each boundary condition set, the corresponding unit the parameters have, and which parameter value was implemented at the corresponding outlet arteries

Set	Name	Parameter	Unit	LACA	RACA	LMCA	RMCA	LPCA	RPCA
1	zero pressure (ZP)	$\bar{p}$	mmHg	87					
2	symmetrical pressure (SP)	$\bar{p}_n$	mmHg	84		86		91	
		c_n	-	20.4		51.05		1	
3	direct experimental pressure (DEP)	(complete data can be found in the Supplementary Dataset 1 online)							
4	Windkessel model (WM)	R_n	mmHg s mL^{-1}	53.90		30.49		83.53	
		C_n	mL mmHg^{-1}	0.0037		0.0065		0.0024	
5	phase modulation (PM)	$\bar{p}_n$	mmHg	84		86		91	
		f	Hz	1.0					
		φ	rad	-0.936					
		β	-	0.625					
		PI_p	-	0.774					

Supplementary Table S3 Arteries at the boundaries of the anatomical model and areas of the interfaces

Artery	Interface area (mm^2)
left internal carotid artery (LICA)	72.0
right internal carotid artery (RICA)	71.9
left vertebral artery (LVA)	17.7
right vertebral artery (RVA)	17.7
left posterior cerebral artery, P2 segment (LPCA)	17.7
right posterior cerebral artery, P2 segment (RPCA)	17.7
left middle cerebral artery, M1 segment (LMCA)	17.7
right middle cerebral artery, M1 segment (RMCA)	17.9
left anterior cerebral artery, A2 segment (LACA)	17.8
right anterior cerebral artery, A2 segment (RACA)	17.9
